# Surface Decoration of Pt Nanoparticles via ALD with TiO_2_ Protective Layer on Polymeric Nanofibers as Flexible and Reusable Heterogeneous Nanocatalysts

**DOI:** 10.1038/s41598-017-13805-2

**Published:** 2017-10-17

**Authors:** Asli Celebioglu, Kugalur Shanmugam Ranjith, Hamit Eren, Necmi Biyikli, Tamer Uyar

**Affiliations:** 1 Institute of Materials Science & Nanotechnology and UNAM–National Nanotechnology Research Center, Bilkent University, Ankara, 06800 Turkey; 2 Electrical and Computer Engineering, University of Connecticut, Storrs, CT 06269-4157 USA

## Abstract

Coupling the functional nanoheterostructures over the flexible polymeric nanofibrous membranes through electrospinning followed by the atomic layer deposition (ALD), here we presented a high surface area platform as flexible and reusable heterogeneous nanocatalysts. Here, we show the ALD of titanium dioxide (TiO_2_) protective nanolayer onto the electrospun polyacrylonitrile (PAN) nanofibrous web and then platinum nanoparticles (Pt-NP) decoration was performed by ALD onto TiO_2_ coated PAN nanofibers. The free-standing and flexible Pt-NP/TiO_2_-PAN nanofibrous web showed the enhancive reduction of 4-nitrophenol (4-NP) to 4-aminophenol (4-AP) within 45 seconds though the hydrogenation process with the degradation rate of 0.1102 s^−1^. The TiO_2_ protective layer on the PAN polymeric nanofibers was presented as an effective route to enhance the attachment of Pt-NP and to improve the structure stability of polymeric nanofibrous substrate. Commendable enhancement in the catalytic activity with the catalytic dosage and the durability after the reusing cycles were investigated over the reduction of 4-NP. Even after multiple usage, the Pt-NP/TiO_2_-PAN nanofibrous webs were stable with the flexible nature with the presence of Pt and TiO_2_ on its surface.

## Introduction

Surface decoration of noble metal nanostructures onto electrospun nanofibrous webs could improve their potential utility over numerous applications such as biosensors^1^, hydrogen sensing^2^ fuel convention^3^, batteries^4,5^, photocatalysis^6,7^ because of its distinctive properties with highly interactive surface area. Nobel metal nanostructures have high interest in the field of catalytic reduction of organic pollutants through the hydrogenation process for the reduction of toxic organic pollutants^8–11^. Research on electron transfer ability from the metal nanostructures by the support or membrane template has improved its activity towards the kinetic exhibition on reaction process for its effective catalytic exhibition^12^. From the family of noble metal ions, platinum (Pt) metal nanostructures has exhibited significant advantages in promising applications by being able to control their size and morphology^13,14^. However, exhibition of reduction performance and its reusability and recoverable functionality of the metal catalyst has emerged as a critical aspect for the catalytic functional properties.

By avoiding the agglomeration and utilizing very low expenditure of metal nanostructures highlights the great impact of the hydrogenation process^15^. The atomic layer deposition (ALD) is a controlled growth process used to decorate or load the monodispersed metal nanoparticles (NP) at a higher growth rate^16^. It has been shown that the ALD process for the decoration of inorganic (metal or metal oxide) nanostructures on the polymeric support materials facilitate to avoid the agglomeration and detachment of nanostructures for the specific purposes^17–19^. More importantly, the surface decoration of metal NP on the flexible polymeric nanofibrous webs will provide the improvised surface area for the interaction and avoid the issues related to the recoverability and multiple usages. However, the two main routes that reduce the catalytic activity and durability of the metal nanostructures over the flexible polymeric membranes are, poor stability of the polymeric substrate during the deposition of metal NP and durability of the metal NP on the polymeric surface which might undergo leaching during the reusable process. Promoting the stability and reusability of the catalyst is an effective process that took a step forward of the industrial commercialization. The leaching of metal NP during the catalytic process was often addressed which reduces it reusability^20^ but the interaction of the metal NP with the template or membrane would enhance its durability. On the other hand, the stability of the flexible polymeric support in the form of nanofibrous web would be the crucial issue during the decoration of metal NP in different ALD reactive atmospheres. By placing the protective layer through ALD on nanostructures and polymeric fibers could improvise its stability and durability under different reactive atmospheres and different reaction temperatures^21,22^. In one of the related study, by ALD process, coating of a passivation AlZnO:Al_2_O_3_ layer over the electrospun Cu nanofibers has enhanced its stability against the oxidation and corrosion^23^. Coating an ultrathin protective layer over the polymeric fibers would reliably solve the issues related to its chemical stability. Providing the protection of metal oxide layer over the polymeric surface, could prevent the few cases such as surface and structural decomposition and improve the interaction of metal nanostructures on the surface. Considering the story behind the metal-metal oxide interaction, TiO_2_ is a reducible metal oxide that can strongly react with the noble metals which has brought on the attention for the applications based on the heterogeneous catalytic reactions^24^. Providing the protective nanolayer over the polymeric nanofibers would exhibit stable and high interactive surface area for the metal NP decoration and the interaction of the metal NP with the metal oxide surface would improve the carrier mobility for favorable catalytic behavior^25,26^.

Here, we report the platinum nanoparticles (Pt-NP) decorated flexible electrospun polymeric (polyacrylonitrile (PAN)) nanofibrous web for the effective catalytic reduction of 4-nitrophenol to 4-aminophenol. Through our optimization, we investigated the effective role of TiO_2_ nanocoating on the electrospun PAN nanofibers for the ALD of Pt-NP catalyst and explored the effective hydrogenation process and improvised stability of the Pt-NP during and after the catalytic reactions. The nanocoating of TiO_2_ as a protective layer over the electrospun polymeric nanofibers was attained with the precise controlled by ALD process^27^. Further, monodisperse Pt-NP were decorated on the surface of TiO_2_-PAN nanofibrous web under the reactive ozone environment by ALD. Here, ALD provides the monodispersive decoration of Pt-NP catalyst and the controlled thin layer of TiO_2_ protective layer over the electrospun polymeric nanofibers for the effective catalytic properties. While testing the catalytic reduction of 4-nitrophenol, Pt-NP decorated TiO_2_-PAN nanofibrous webs were exhibited pronounceable performance with the effect of TiO_2_ protective layer. Additionally, the protective layer has influences the higher stability and higher catalytic activity with faster reduction rate for the nanofibrous web. The decoration of metal NP catalyst onto flexible and high surface area nanofibrous substrates has the advantages due to the several factors such as (i) avoid the agglomeration of metal NP, (ii) enhanced catalytic performance along with easy recovery and reusability, (iii) persistent surface level integration with the atomic level functionalities, and (iv) low consumption of catalyst for the reactions.

## Results

### Fabrication of nanofibrous webs

Initially, different polymeric nanofibrous webs such as polyacrylonitrile (PAN), Nylon 66, polysulfone (PSU) were prepared through the electrospinning process and used a high surface area substrate for the deposition of platinum nanoparticles (Pt-NP) through the atomic layer deposition (ALD). The electrospun polymeric nanofibrous webs have uniform fiber morphology, yet, while depositing the Pt through ALD, the morphological studies reveal that Pt deposition through the ALD under ozone atmosphere cause some degradation to the polymeric webs. The electrospun Nylon 66 and PSU nanofibrous webs were highly affected by the ozone environment that started to etched under this reactive atmosphere of ALD (Fig. S1). Even though the PAN nanofibers were stable under the ozone environment, the ALD of Pt nanostructures onto PAN nanofibers were not successful. Hence, to improve the stability, structural protectively and surface interactivity, a thin layer of ZnO, Al_2_O_3_ and TiO_2_ were deposited over the PAN nanofiber surface through the ALD process. While depositing the Pt in ozone environment into the metal oxides coated PAN nanofiber surface, for ZnO-PAN nanofibrous web, we found out that ALD of Pt was not successful. For Al_2_O_3_-PAN nanofibrous web, Pt was deposited but it has majorly exhibited the Pt with Pt^II^ states. But inducing the TiO_2_-PAN nanofibrous web as a substrate for the Pt depositing, it exhibits the deposition of Pt with the promising metallic nature (Fig. S2). Through this optimization, the effective role of TiO_2_ as a protective layer and interactive sites that offered by the TiO_2_ for the Pt deposition onto the PAN nanofibers and its effective role for the catalytic reduction were investigated as below.

**Figure 1:**
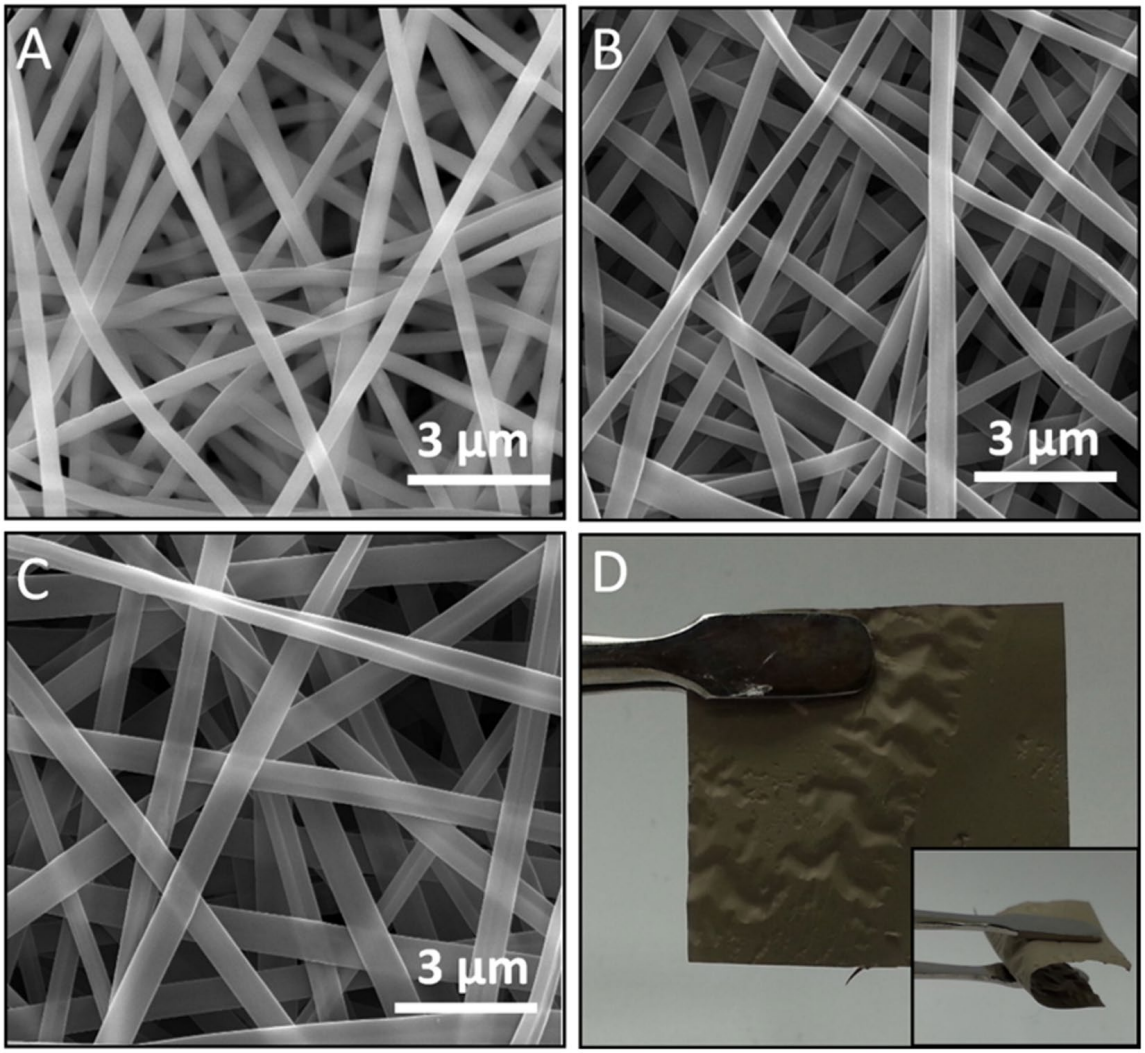
Morphological characterization of nanofibrous webs. Representative SEM images of (**A**) as-electrospun PAN NF, (**B**) TiO_2_-PAN NF and (**C**) Pt-NP/TiO_2_-PAN NF. (**D**) Optical image of Pt-NP/TiO_2_-PAN NF. Before and after the deposition of TiO_2_ and Pt nanostructures, the fiber morphology was quite stable and inset show the flexible nature nanofibrous web.

The as-electrospun PAN nanofibers (NF) reveal the bead-free and homogenous morphology (Fig. 1A). In physical appearance, PAN nanofibrous webs were quite resistant under the reactive ozone atmosphere of ALD of Pt-NP, however, the magnified transmission electron microscopy (TEM) studies exhibit its surface deformation and poor loading of the Pt-NP on the nanofiber surface (Fig. S3). In order to stabilize surface of the polymeric nanofibrous substrate and improvise the interaction of Pt metal ions over the fiber surface, ALD of thin layer of TiO_2_ was coated on the as-electrospun PAN nanofibers (TiO_2_-PAN NF) and after that, the ALD of Pt-NP in ozone atmosphere was performed to produce Pt-NP surface decorated nanofibers (Pt-NP/TiO_2_-PAN NF). Ozone would be a reactive gas on the polymeric surface and it would start to etch the surface and exhibit poor surface interaction of Pt-NP with the polymeric surface. By providing an ALD of thin layer of TiO_2_ coating on the PAN nanofibers, the surface stability and the interaction of Pt-NP on the surface of the nanofibers was improved. TiO_2_ and Pt-NP were deposited through the ALD process over the electrospun PAN nanofibers and SEM images (Fig. 1) show that before and after the deposition of TiO_2_ and Pt nanostructures, the fiber morphology was quite stable. The change in color (from white (PAN NF) to yellowish (TiO_2_-PAN NF) and then dark brown (Pt-NP/TiO_2_-PAN NF)) of the nanofibrous web after the ALD deposition confirms the deposition of TiO_2_ layer and Pt-NP/TiO_2_ layer over the polymeric nanofibers (Figs 1 and S4). The nanofibers exhibited uniform size distribution with average diameter around 360–400 nm, 410–450 nm and 550–570 nm for the pristine PAN NF, TiO_2_-PAN NF and Pt-NP/TiO_2_-PAN NF, respectively (Fig. S5). The slight changes in fiber diameter of TiO_2_-PAN NF and Pt-NP/TiO_2_-PAN NF were possibly due to some flattening of the fiber morphology during the ALD process.

### Characterization of the nanofibrous webs

**Figure 2:**
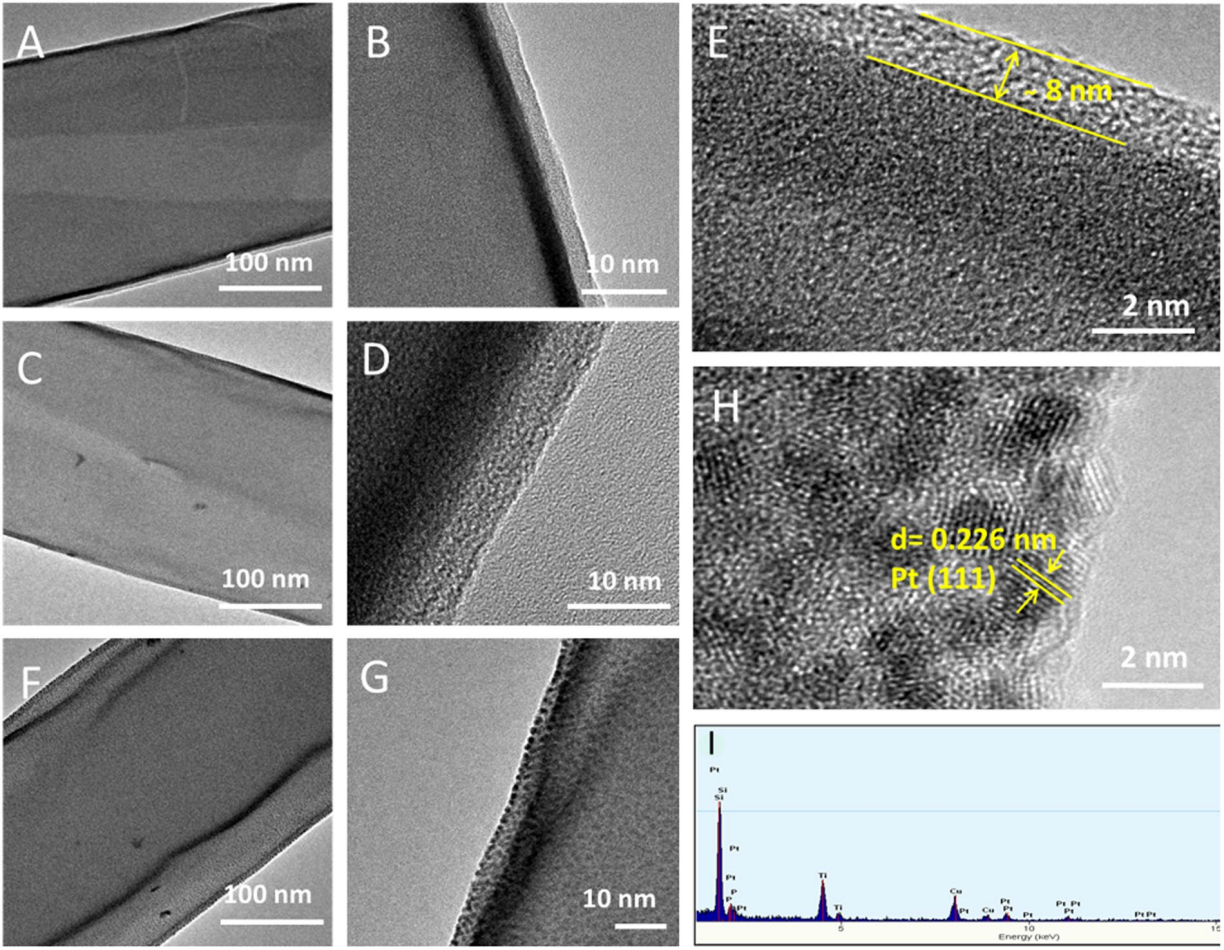
TEM, HRTEM and EDAX characterization of Pt-NP/TiO_2_-PAN NF. (**A**,**B**) TEM and HRTEM of the PAN NF, (**C**–**E**) TEM and HRTEM of the TiO_2_-PAN NF, (**F**–**H**) TEM and HRTEM of the Pt-NP/TiO_2_-PAN NF and (**I**) EDAX spectra of the Pt-NP/TiO_2_-PAN NF. The fiber surfaces were decorated with the monodispersed Pt-NP with the size around ~2 nm. EDAX spectra reveal the presence of Ti and Pt in the Pt-NP/TiO_2_-PAN NF confirming the presence of Pt-NP over the TiO_2_-PAN NF.

To denote the size distribution of Pt-NP and layer thickness of the TiO_2_ protective layer, TEM and HRTEM imaging was carried out for pristine PAN NF, TiO_2_-PAN NF and Pt-NP/TiO_2_-PAN NF (Fig. 2). After 150 cycles of ALD, nearly 8 nm thickness of TiO_2_ nanocoating formed over the Si reference wafer. After this optimization study, 150 cycles of ALD of TiO_2_ over the PAN nanofibers was applied in order to have TiO_2_-PAN NF samples having approximately 5–8 nm thickness of TiO_2_ based protective layer. After the ALD of TiO_2_, there was no other notable change on the PAN NF except the slight increase in fiber diameter, with the preservation of fiber morphology (Fig. 2C,D). The HRTEM images of the TiO_2_-PAN NF reveal that the TiO_2_ surface was in amorphous states which has closely integrated with the polymeric surface. Moreover, the absence of diffraction peak of TiO_2_ for the TiO_2_-PAN NF sample reveals the amorphous nature of the TiO_2_ protective layer (Fig. S6). Further ALD of Pt over the TiO_2_-PAN NF led to the interaction of Pt over the fiber surface and formation of the Pt-NP decorated flexible nanofibrous membranes (Pt-NP/TiO_2_-PAN NF). TEM and HRTEM images (Fig. 2F–H) reveal that the fiber surfaces were decorated with the monodisperse Pt-NP with a size around ~2 nm. The individual Pt grains clearly evidence the (111) facet orientation of single crystalline Pt nanograin functionality on the nanofiber surface with the lattice spacing of 0.226 nm. Because of this ultra-small size, we were not able to characterize the presence of Pt-NP from the X-ray diffraction (XRD). Yet, the EDAX spectra reveal the presence of Ti and Pt in the Pt-NP/TiO_2_-PAN NF confirming the presence of Pt-NP over the TiO_2_-PAN NF (Fig. 2I). The functional groups of the polymeric surface promote the nucleation of TiO_2_ on the surface^28^, further, the over coating of TiO_2_ permit the strong interaction for the metal-metal oxide, offering the platform for Pt-NP decoration by ALD. Without the protective TiO_2_ coating, Pt did not interact with the PAN fiber surface and the ozone reactive environment cause some deformation to the PAN polymeric fibrous structure.

**Figure 3:**
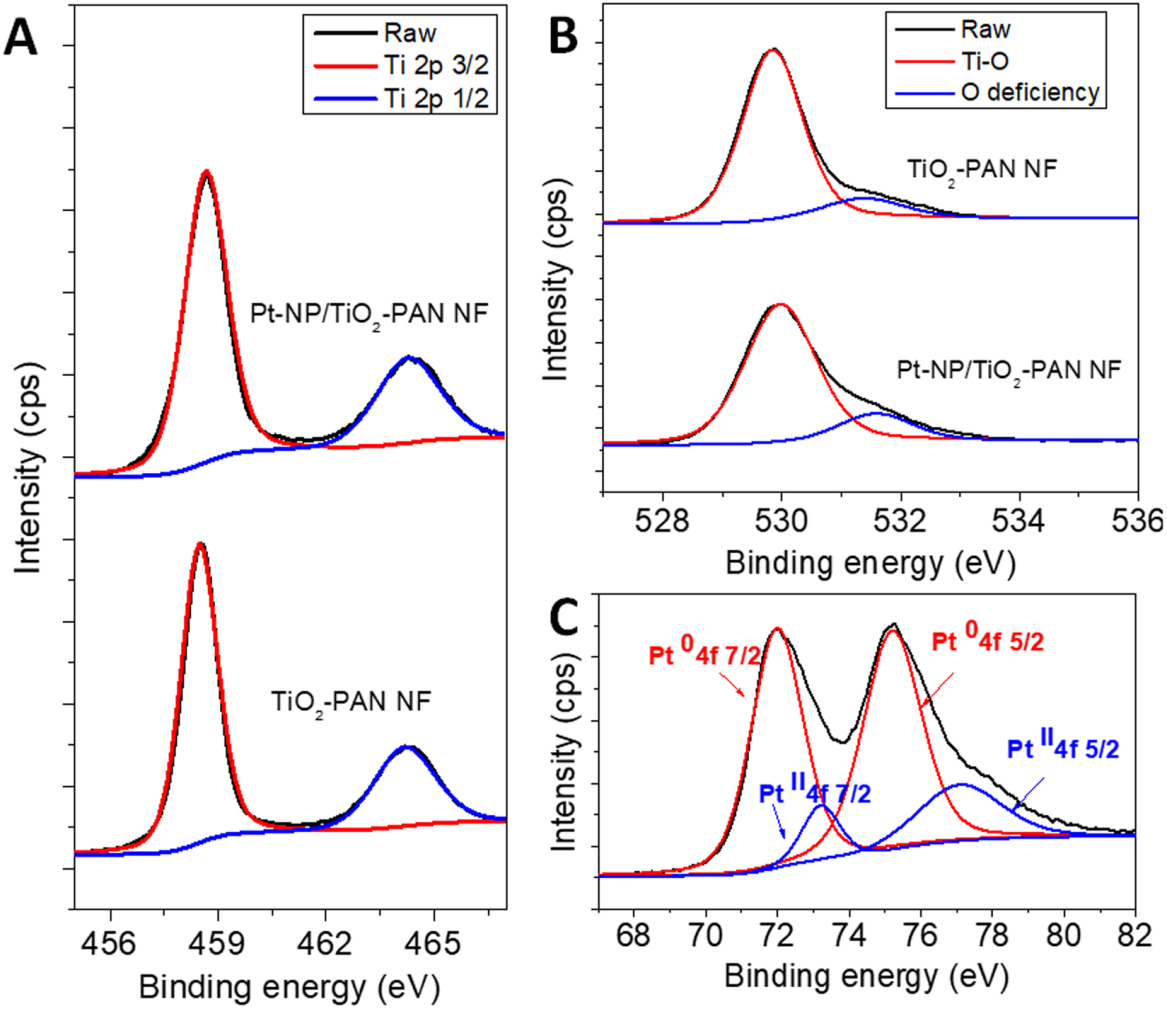
Structural characterization of nanofibrous webs. High resolution XPS spectra of TiO_2_-PAN NF and Pt-NP/TiO_2_-PAN NF: (**A**) Ti 2p; (**B**) O 1 s; (**C**) Pt 4 f. The two distinctive spectra of the Ti 2p 3/2 and Ti 2p 1/2 peaks at 458.49 eV and 464. 31 eV confirming the presence of Ti in the oxidized state over the PAN polymeric surface. Calculated stoichiometric ratio of Ti/O from the spectra and the lattice oxygen vibration with the metal oxide attributed at the 529.9 eV provide evidence for the formation of Ti with the oxidized state. While observing the high resolution scan of Pt 4 f region, Pt exhibited promising metallic states (Pt^0^ oxidation state) with nearly 20.21**%** of Pt^II^ oxidation states.

XPS analyses reveal the elements present and its structural nature over the PAN NF. Figure S7 shows the survey spectrum of the Pt-NP/TiO_2_-PAN NF web which confirms the presence of Ti, O, and Pt over the polymeric surface and the Table S1 reports the weight percentage of Pt, Ti, O and C in this flexible nanofibrous web sample. The high resolution XPS scan of Ti 2p, O 1 s and Pt 4 f regions are exhibited in the Fig. 3. Figure 3A shows two distinctive spectra of the Ti 2p 3/2 and Ti 2p 1/2 peaks at 458.49 eV and 464. 31 eV confirming the presence of Ti in the oxidized state over the PAN polymeric surface^29^. Calculated stoichiometric ratio of Ti/O from the spectra and the lattice oxygen vibration with the metal oxide attributed at the 529.9 eV (Fig. 3B) provide evidence for the formation of Ti with the oxidized state^30^. While observing the high resolution scan of Pt 4 f region (Fig. 3C), Pt exhibited promising metallic states (Pt^0^ oxidation state) with nearly 20.21% of Pt^II^ oxidation states which was clearly correlated with the Si reference substrate during the deposition (Fig. S8). It is worth to note that after the TiO_2_ protective layer deposition, Pt decoration exhibited the zero oxidation state, which features the enhanced local electron density by the effective charge transfer from the TiO_2_ to Pt results from the metal-metal oxide interaction^31^. FTIR studies reveal that after the ALD process, the chemical structure of the PAN polymer is still protected, since the vibration spectrum of -CN triple bond nature exhibited at the ~2300 cm^−1^ was promisingly stable after the ALD process also, which signifies the stability of the PAN after the ALD of TiO_2_ layer and Pt-NP decoration (Fig. S9). Electrospun PAN nanofibrous webs have shown thermal stability up to ~320 °C which reveal the stability of the polymeric web during the ALD process and the protective coating of TiO_2_ was influenced further on improvised thermal stability which has confirmed by the TGA thermograms (Fig. S10).

### Catalytic reduction of 4-nitrophenol (4-NP)

**Figure 4:**
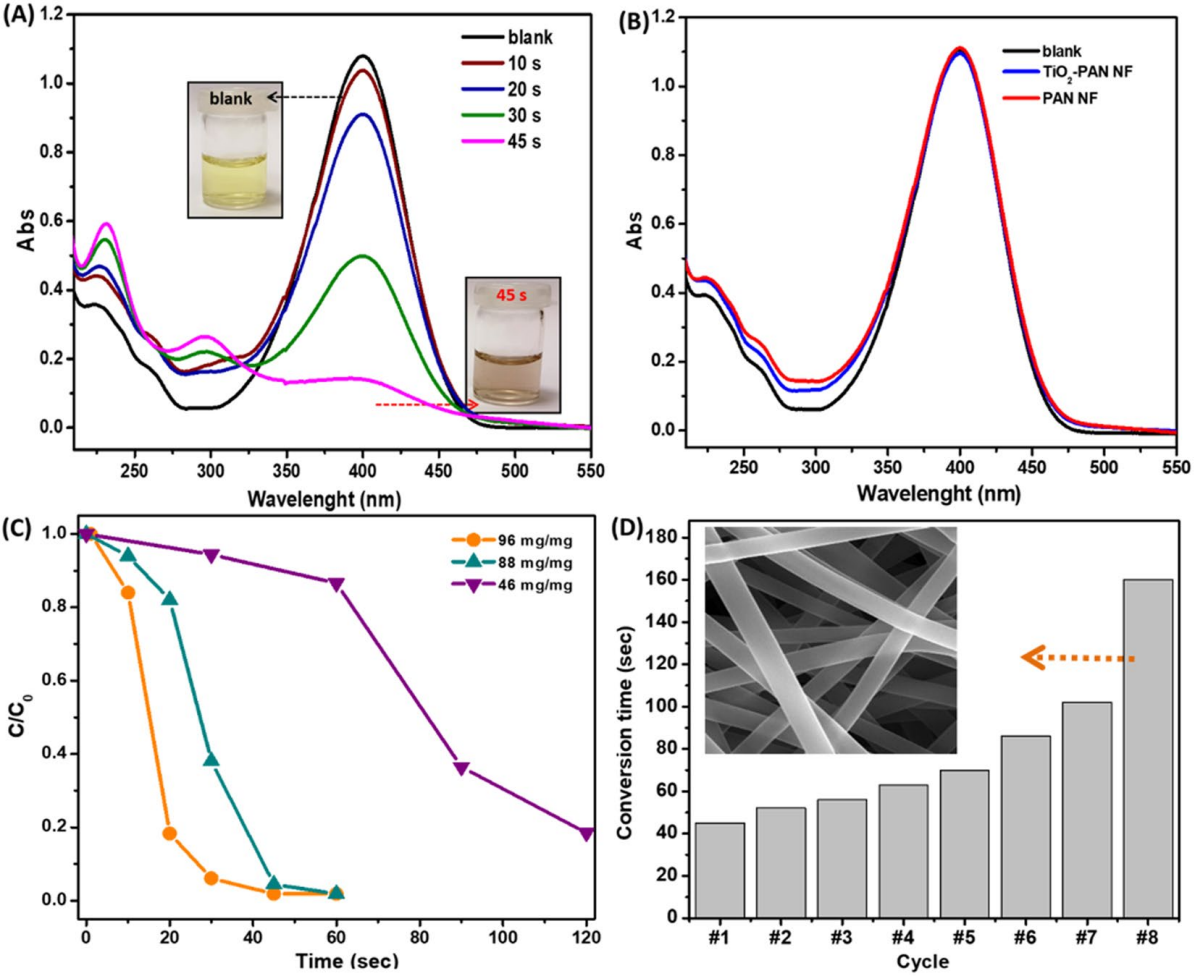
Catalytic reduction of 4-nitrophenol (4-NP). (**A**) Time dependent 4-NP reduction test on using Pt-NP/TiO_2_-PAN NF as a catalyst nanofibrous web in 45 sec. Insert shows the color change of 4-NP solution in a glass vial with the catalytic Pt-NP/TiO_2_-PAN NF after 45 sec. (**B**) Absorption spectra of 4-NP on using TiO_2_-PAN NF and PAN NF after the time period of 5 minutes. (**C**) Time dependent reduction of 4-NP with the different dosages of Pt-NP/TiO_2_-PAN NF catalyst ratio with 4-NP. (For instance; 96 mg/mg means that for 1 mg 4-NP we used 96 mg Pt-NP/TiO_2_-PAN NF sample). (**D**) Reusable performance of Pt-NP/TiO_2_-PAN NF as a catalyst web for consecutive cycles and (inset) SEM image of nanofibrous web after 8^th^ cycle catalytic test. (For reusability test NF/4-NP ratio was 100 mg/mg).

To evolve the effective catalytic behavior with the impact on the protective TiO_2_ layer, the reduction of 4-nitrophenol (4-NP) was monitored and presented in the Fig. 4. The time dependent reduction of 4-NP by Pt-NP/TiO_2_-PAN NF having weight ratio of 96 mg/mg (using 96 mg of catalyst for the 1 mg of 4-NP) is shown in the Fig. 4A. The rapid decrease in the absorption intensity of the 4-nitrophenolate at 400 nm and the appearance of the spectral absorption at 298 nm, which denoted the 4-aminophenol (4-AP), signifies the reduction of 4-NP to 4-AP with the presence of metal catalyst. The faster reduction behavior was observed within 45 sec (Fig. 4A) with the presence of Pt-NP/TiO_2_-PAN NF based catalyst and the inset shows the change in the color of 4-NP solution to 4-AP solution during the reduction process. Here, while immersing the Pt-NP/TiO_2_-PAN NF web into the 4-NP:NaBH_4_ mixture solution with the continuous shaking, 4-NP was reduced to 4-AP through the feasible interaction with Pt-NP which facilitates the electron transfers from the BH^4−^ ions to the 4-NP, which is necessary for the reduction process^32,33^. Mixing the 4-NP with the NaBH_4_ favor the formation of 4-nitrophenolate component, which was denoted by the shifted peaks at 400 nm in the absorption spectra. With the presence of nanofibrous web catalyst, the progress of the reaction was monitored by the change in absorption spectral intensity at 400 nm.

For observing the control for the reaction, the as-electrospun PAN NF and TiO_2_-PAN NF were subjected to the catalytic reaction (Fig. 4B) and the absorption peaks at 400 nm remained unchanged even after 30 min which confirms the Pt-NP role over the polymeric nanofibrous web in catalytic reaction for Pt-NP/TiO_2_-PAN NF sample. It’s important to note that the weight ratio of the catalyst play a significant role for the accelerating the reduction process. We attained the reduction of catalytic activity with three different weight ratio of catalytic web with the constant 4-NP dosage. Figure 4C shows that under the weight ratio of 96 mg/mg, the reduction was much faster (within 60 sec) compared to lower weigh ratio (46 mg/mg). While varying the catalyst weight percentage with the 4-NP ratio, the reduction rate changed which signifies faster degradation rate (0.1102 s^−1^) on increasing the amount of Pt-NP/TiO_2_-PAN NF and in the lower ratio (46 mg/mg), inability to reduce the pollutant completely. From the ICP-MS results, the loading of Pt-NP over the polymeric nanofibrous web was nearly 63 µg/mg which shows very low consumption of Pt-NP catalyst for the catalytic reduction. During the first cycle reaction process, it has leached around 1.18 ng/mg which signifies the durability of the metal catalyst over the polymeric surface. The reduction rate of Pt NP/TiO_2_-PAN NF nanofibrous web as catalytic work effectively completed in 45 sec (for 100 mg/mg sample), even though the presence of only 63 µg/mg of metal catalyst in the web structure, whereas rGO@Pd@C^34^, Au-graphene^35^, Au@C^36^, Ag/Carbon fibers^37^ catalyst are finished after 5 mins (Table 1). Further, turnover frequency (TOF), defined as mole of the reactant (4-NP) converted by per mole of active metal in catalyst per minute, also shows much higher TOF values for Pt NP/TiO_2_-PAN NF when compared to some of the previous reports (Table 1). Figure 4D shows the reusability of the Pt-NP/TiO_2_-PAN NF catalysts nearly eight consecutive cycles. The catalytic Pt-NP/TiO_2_-PAN NF exhibited well stability up to 7 cycles of reaction with 100% conversion within 100 sec reaction periods. On utilizing the catalytic membrane in multiple cycles, it has exhibit the reduction of 4-NP but the reduction rate has reduced with respect to the consecutive recycles. Inset of the Fig. 4D shows that, after the reusable process, the morphology of the nanofiber remain unchanged which exhibit the potential role of TiO_2_ coating to retain the structural stability of the polymeric surface in the reduction process. After the multiple usage in reduction process, the XPS results of the Pt-NP/TiO_2_-PAN NF nanofibrous web reveal that the weight ratio of the Pt ion reduced without changing its chemical structure (Fig. S11). XPS results reveal the detachment of Pt-NP from the nanofibrous membrane which causes the slight decrease in the catalytic reduction rate after every consecutive recycles. But even after three cycles, Pt-NP/TiO_2_-PAN NF still exhibit more than 50% reduction efficiency.

**Table 1:** Comparison for the reduction of 4-NP with different catalysts.

Catalyst	Mass of the catalyst (mg)	Amount of 4-NP (mM)	Amount of NaBH_4_ (mM)	Metal size (nm)	Metal content (wt %)	Conversion time (sec)	TOF	Stability cycles	Ref
Pt-NP/TiO_2_-PAN NF	2	1 × 10^−1^	7	2	6.3	45	4.44	8	Present work
rGO@Pd@C	5	3 × 10^−4^	3 × 10^−2^	4	0.28	30	4.56	10	34
Au/Graphene	0.1	2.8 × 10^−4^	2 × 10^−2^	14.6	24	720	0.19	1	35
Au@C	5	3 × 10^−4^	3 × 10^−2^	15	NA	300		5	36
Ag/Carbon nanofiber	1	3.6 × 10^−3^	15 × 10^−2^	28.1	8.4	480	0.58	3	37

**Figure 5:**
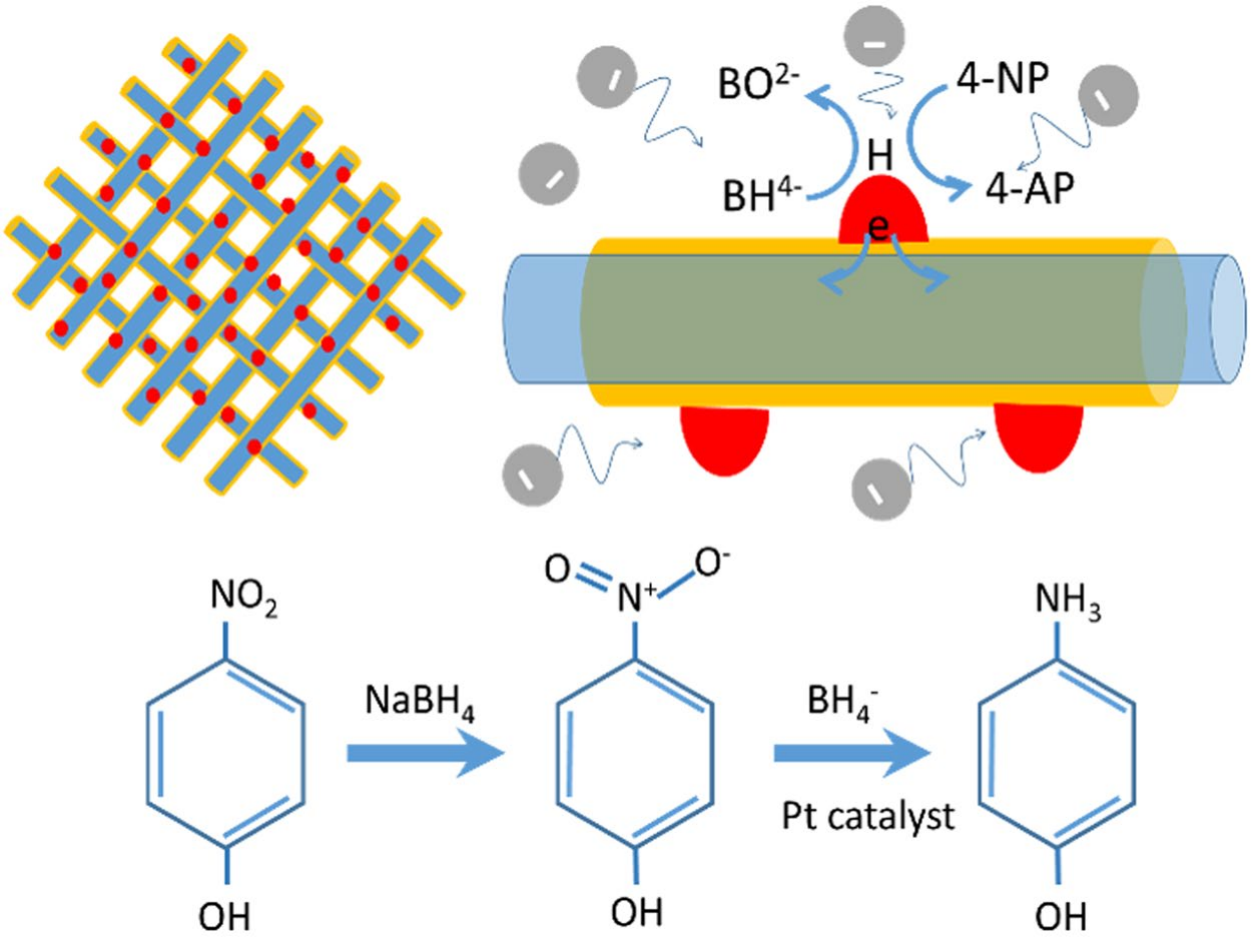
Schematic illustration of the synergistic catalytic mechanism for the 4-NP reduction over Pt-NP/TiO_2_-PAN NF. BH_4_^−^ ion reduces the 4-NP by the effective transfer of hydrogen from the BH_4_^−^ to 4-NP through the metal catalyst. The interaction at the interface of the Pt and TiO_2_ on the nanofiber surface migrate few electron to the protective layer (TiO_2_) through the metal support interaction which make the metal catalyst as electron deficient. In this condition, negatively charged 4-nitrophenolate and BH_4_^−^ preferably interact with the metal catalyst and facilitate the hydrogenation process on the 4-nitrophenolate. Reactants have to be absorbed on the catalytic surface and the BH_4_^−^ will donate the electrons to the electron deficient catalytic surface which activate the hydrogen atoms through the fissure of B-H bond on the Pt surface. The thermodynamically unstable hydrogen atoms will react with the 4-nitrophenolate through the hydrogenation process and is reduced to 4-AP.

Here, the protective TiO_2_ layer plays an effective role initiates the Pt to interact with the nanofiber surface for preparing the flexible catalytic nanofibrous web. With the presence of very low consumption of Pt NP, it is not anticipated to observe such faster catalytic behavior. The TiO_2_ protective layer plays a big role for speeding up the catalytic reduction process. Our proposed mechanism elucidate the promising catalytic behavior by the Pt/TiO_2_ interface at the fiber surface (Fig. 5). In general, it is believed that BH_4_^−^ ion reduce the 4-NP by the effective transfer of hydrogen from the BH_4_^−^ to 4-NP through the metal catalyst^12^. The interaction at the interface of the Pt and TiO_2_ on the fiber surface migrate few electron to the protective layer (TiO_2_) through the metal support interaction which make the metal catalyst as electron deficient. In this condition, negatively charged 4-nitrophenolate and BH_4_^−^ preferably interact with the metal catalyst and facilitate the hydrogenation process on the 4-nitrophenolate. As per the schematic illustration, reactants has to be absorbed on the catalytic surface and the BH_4_^−^ will donate the electrons to the electron deficient catalytic surface which activate the hydrogen atoms through the fissure of B-H bond on the Pt surface. The thermodynamically unstable hydrogen atoms will react with the 4-nitrophenolate through the hydrogenation process and is reduced to 4-AP. The nanofibrous substrate provided the favorable interaction for the hydrogenation process on the catalytic surface presenting it as a promising catalytic membrane.

## Discussion

We fabricated the Pt-NP based flexible nanofibrous catalyst for the reduction of 4-NP through the electrospinning process followed by the ALD. Thin layer of TiO_2_ was over coated on the electrospun polymeric nanofibers for improving the polymer stability and to enhance the decoration of Pt-NP by ALD on the high surface area nanofibrous substrate. Monodispersed Pt-NP with the size around ~2 nm were decorated on the surface of TiO_2_-PAN NF by ALD (Pt-NP/TiO_2_-PAN NF) and the catalytic property of this flexible and free-standing high surface area nanofibrous web was investigated. The TiO_2_ protective layer on the PAN polymeric nanofibers was presented as an effective route to enhance the attachment of Pt-NP and to improve the structure stability of polymeric nanofiber substrate. Presence of metal-metal oxide interaction over the nanofiber surface has enhanced the functional properties of this flexible nanofibrous web. The catalytic reduction rate was varied on changing the dosage (48, 88, 96 mg/mg) of Pt-NP/TiO_2_-PAN NF in the reaction and also the nanofibrous membrane exhibit notable reusable catalytic reduction of 4-NP to 4-AP for several consecutive cycles without change in its morphology.

## Methods

### Materials

Polyacrylonitrile (PAN, Mw ∼150000, Scientific Polymer Products, Inc), Nylon 66 (Mw 230000–2800000, Scientific Polymer Products, Inc), Polysulfone (PSU, Scientific Polymer Products, Inc), N, N- dimethylformamide (DMF, Pestanal, Riedel), Formic acid (HCOOH, 98%, Sigma-Aldrich), N,N- dimethylacetamide (DMAc, 99%, Sigma-Aldrich), Acetone (>99%, Sigma-Aldrich), 4-nitrophenol (4-NP, 99%, Alfa Aesar), sodium borohydride (NaBH_4_, fine granular, Merck) were obtained commercially. All materials were used without any purification. De-ionized (DI) water is obtained from Millipore Milli-Q system.

### Electrospinning

To obtain uniform and bead-free nanofibrous web, we have prepared homogeneous PAN, Nylon 66 and PSU in DMF, formic acid and DMAc:acetone (9:1, v-v) at 13%, 8% and 32% (w/v, with respect to solvent) polymer concentrations, respectively. After that, the well-stirred solution was loaded in 3 mL or 10 mL syringe fitted with a metallic needle of 0.4 mm inner diameter. The syringe was located horizontally on the syringe pump (KD Scientific, KDS 101) with a 0.5–1 mL/h flow rate. A high voltage; 10–15 kV was applied by high voltage power supply (Matsusada, AU Series) to the tip of the needle to initiate the electrospinning jet movement through the stationary plate metal collector which is covered with aluminum foil and positioned at 10 cm from the end of the tip. The electrospinning process was carried out at ∼25 °C and 22% relative humidity in an enclosed Plexi-glass chamber. The all collected nanofibers/nanowebs were place in the hood for overnight to remove the residual solvent.

### Atomic layer deposition (ALD)

ALD technique was employed to PAN nanofibrous web for the coating of TiO_2_ protective layer and decoration of Pt-NP on TiO_2_-PAN nanofibers. Firstly, TiO_2_ layer was coated by using Savannah S100 ALD reactor (Ultratech Inc.) as a protecting layer against the ozone exposure during the ALD of Pt-NP. The substrate temperature was kept at 150 °C during the ALD process using Ti(NMe_2_)_4_ and H_2_O as titanium and oxygen precursors, respectively. Prior to deposition, Ti(NMe_2_)_4_ precursor was preheated to 75 °C and as the carrier gas, N_2_ was used with a flow rate of 20 sccm. The deposition was carried out using 150 deposition cycles of TiO_2_ with the estimated growth rate ∼0.44 Å/cycle onto the PAN nanofibers. Subsequently, Pt nanoparticles were decorated onto TiO_2_-PAN nanofibers by using trimethyl (methylcyclopentadienyl) platinum (IV) (MeCpPtMe_3_) as Pt precursor and O_3_ as counter reactant. The temperature of Pt precursor was held at 65 °C to obtain a proper vapor pressure. O_3_ was produced from a pure O_2_ flow with a Cambridge NanoTech Savannah Ozone Generator. ALD of Pt was carried out at 150 °C as well. During the optimization part, electrospun PSU and Nylon nanofibers were also coated with Pt precursor by using the same procedure above. Additionally, ZnO and Al_2_O_3_ layers were coated on polymeric nanofibers as protecting layer just before the Pt-NP decoration. During the ZnO and Al_2_O_3_ coating process, the substrate temperature was arranged as 150 °C by using ZnEt_2_ and Al(CH_3_)_3_ precursor, respectively. For both of them, H_2_O was used as oxygen precursors, N_2_ as carrier gas with a flow rate of 20 sccm. While, the deposition of ZnO was carried out by using 50 deposition cycles with the estimated growth rate of ∼1.48 Å/cycle, these values are kept as 75 and ∼1.01 Å/cycle during the Al_2_O_3_ coating.

### Characterization

The morphology of the nanofibrous webs was studied by using scanning electron microscope (SEM, FEI-Quanta 200 FEG). The nanofibers were sputter coated with 5 nm Au/Pd prior to SEM measurements. These images were used to calculate the average fiber diameter of nanofibers. Transmission electron microscopy (TEM) was used to detect the Pt-NP on the nanofiber surface. For this, samples were dispersed in ethanol by using vortex and then small amount of this dispersion is dropped on the porous carbon coated TEM grid. TEM (FEI-Tecnai G2F30) and elemental analysis (energy dispersive X-ray spectroscopy, EDX) was carried out for Pt-NP/TiO_2_-PAN nanofibers. The surface analyses of the samples were performed by using X-ray photoelectron spectroscopy (Thermoscientific, k-Alpha) under Al Kα (hυ = 1486.6 eV) line with a charge neutralizer. Pass energy, step size and spot size were 30 eV, 0.1 eV and 400 µm, respectively. Avantage software was used for the deconvolution of peaks. X-ray diffraction (XRD) patterns from the PAN, TiO_2_-PAN and Pt-NP/TiO_2_-PAN nanofibrous web were collected (2θ = 10°–90°) by using PANalytical X’Pert Pro MPD X-ray Diffractometer Cu Kα radiation. Fourier transform infrared spectrometer (FTIR) (Bruker-VERTEX 70) was used to record the infrared spectra of PAN and Pt-NP/TiO_2_-PAN nanofibrous web. For measurement, the samples were mixed with potassium bromide (KBr) and pressed as pellets. The scans (64 scans) were recorded between 4000 cm^−1^ and 400 cm^−1^ at a resolution of 4 cm^−1^. Thermogravimetric analysis (TGA, TA Q500, USA) was carried out to determine the thermal properties of the PAN, TiO_2_-PAN and Pt-NP/TiO_2_-PAN fibrous webs from 25 to 600 °C with a heating rate of 20 °C min^−1^ under nitrogen gas flow. Inductively coupled plasma mass spectrometry (ICP-MS) was used to detect the Pt-NP amount in the composite structure and the leaching amount of Pt-NP through the liquid environment after catalyst test. For this, samples were dissolved in HF: HCl: HNO_3_ solution. The samples were diluted to 10 ml of 2% HCl solution. Platinum standards of 250 ppb, 125ppb, 62.5 ppb and 31.25 ppb were prepared in 2% HCl solution and 2% HCl solution was used as blank. Thermo X series II inductively coupled plasma-mass spectrometer was used to accomplish the measurements. The ICP-MS operating parameters were: dwell time-10 000 ms, channel per mass^−1^, acquisition duration-7380, channel spacing-0.02, carrier gas-argon.

### Catalytic reduction of 4-nitrophenol (4-NP)

The catalytic performance of nanofibrous web was investigated by the reduction of 4-nitrophenol (4-NP) to 4-aminophenol (4-AP) along with the existence of sodium borohydride (NaBH_4_). Firstly, the aqueous solutions of both 4-NP (0.1 mM) and NaBH_4_ (7 mM) are mixed at the required ratio. Afterwards, Pt-NP/TiO_2_/PAN nanofibrous web (96 mg/mg (nanofiber/4-NP)) was immersed in this blend solution and shaken at 320 rpm. Meanwhile, the catalytic reaction was monitored with the progressing time at 400 nm by using UV-Vis spectrophotometer (Varian, Carry 100). For comparison, the same experiment was also performed for PAN and TiO_2_-PAN nanofibrous web. The catalytic performance of Pt-NP/TiO_2_-PAN nanofibrous web was also evaluated according to sample amount. Therefore, 96, 88 and 46 mg/mg (nanofiber/4-NP) systems were exposed to same experiment procedure. All the experiments were done twice and the mean value has taken for quantifying the degradation rate. Finally, the reusability performance of Pt-NP/TiO_2_-PAN nanofibrous web was checked by applying a slight washing step to the nanoweb in water after each catalyst reaction, and then nanowebs are dried and used for the next cycle. After reusability test, the morphology of nanofibers was investigated by SEM technique.

### Data availability

All data are available from the authors on reasonable request.

## Electronic supplementary material

Supporting Information
