# Supplementary material for: Surface Decoration of Pt Nanoparticles via ALD with TiO2 Protective Layer on Polymeric Nanofibers as Flexible and Reusable Heterogeneous Nanocatalysts
Source: Sci Rep. 2017 Oct 17;7:13401. doi: 10.1038/s41598-017-13805-2 (PMC5645354; doi:10.1038/s41598-017-13805-2)
Supplement: Supplementary file 1 — Supporting Information [file 41598_2017_13805_MOESM1_ESM.pdf]

Supportive Information

Surface Decoration of Pt Nanoparticles via ALD with TiO_2 Protective Layer on Polymeric Nanofibers as Flexible and Reusable Heterogeneous Nanocatalysts

Asli Celebioglu[a+], Kugalur Shanmugam Ranjith[a+], Hamit Eren[a], Necmi Biyikli[b], Tamer Uyar[a]*.

[a] Institute of Materials Science & Nanotechnology and UNAM–National Nanotechnology Research Center, Bilkent University, Ankara, 06800, Turkey.

[b] Electrical and Computer Engineering, University of Connecticut, Storrs, CT 06269-4157, USA

*Corresponding Author: T.U. (email: uyar@unam.bilkent.edu.tr)
+ Equal Contribution

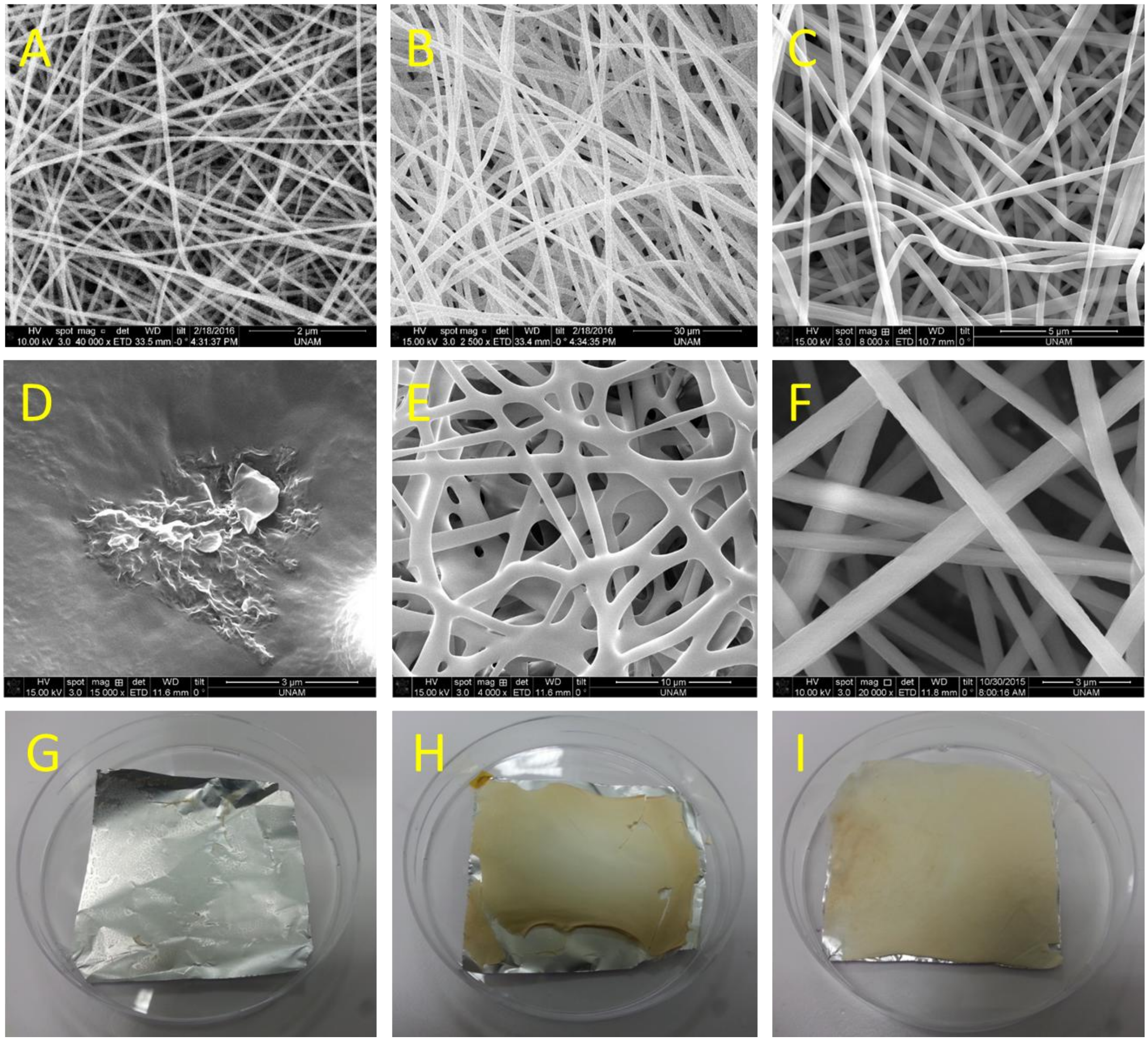

Fig. S1 SEM and the optical images of the as-electrospun polymeric nanofibrous webs (A) Nylon 66, (B) PSU, (C) PAN, and Pt deposited electrospun nanofibrous webs (D, G) Pt deposited Nylon 66, (E, H) Pt deposited PSU and (F, I) Pt deposited PAN nanofibrous web.

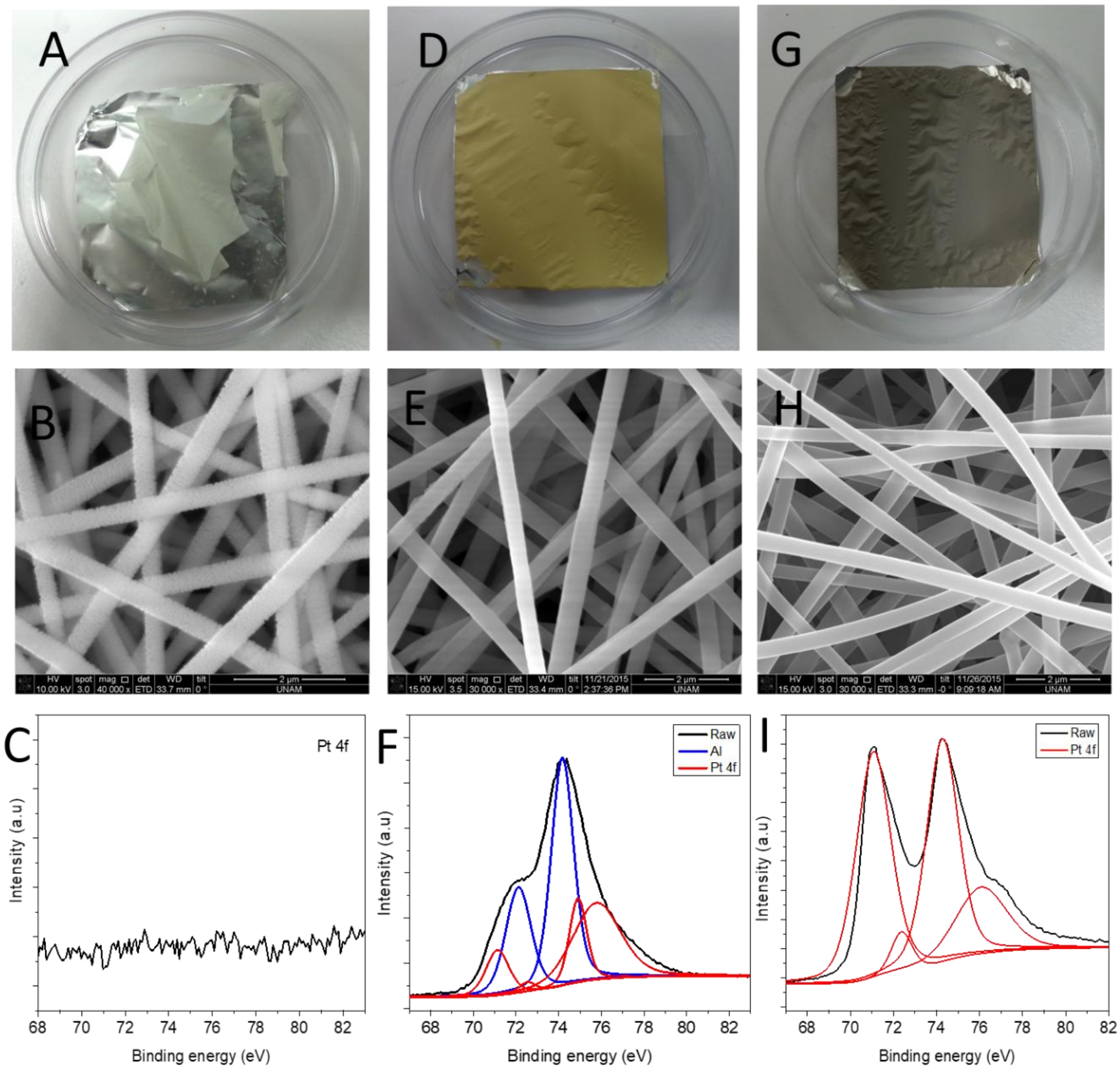

Fig. S2 Optical, SEM images and high resolution Pt 4f XPS spectra of the 15 cycles of Pt deposited electrospun nanofibrous web samples: (A, B, C) ZnO-PAN, (D, E, F) Al_2O_3-PAN and (G, H, I) TiO_2-PAN.

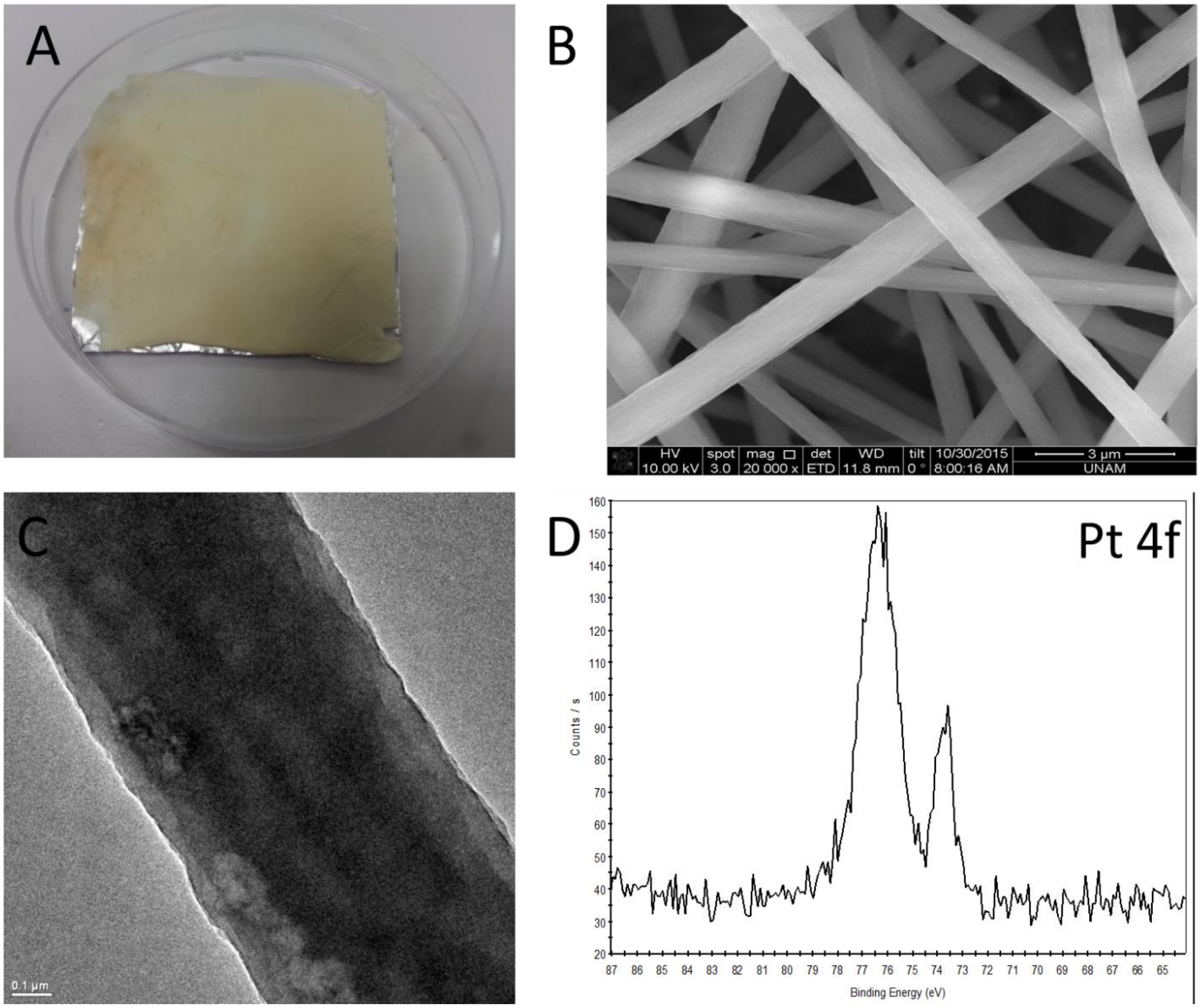

Fig. S3 Optical, SEM and TEM and high resolution Pt 4f state XPS spectra of the 15 cycles of Pt deposited through the ALD on the electrospun PAN nanofibrous web.

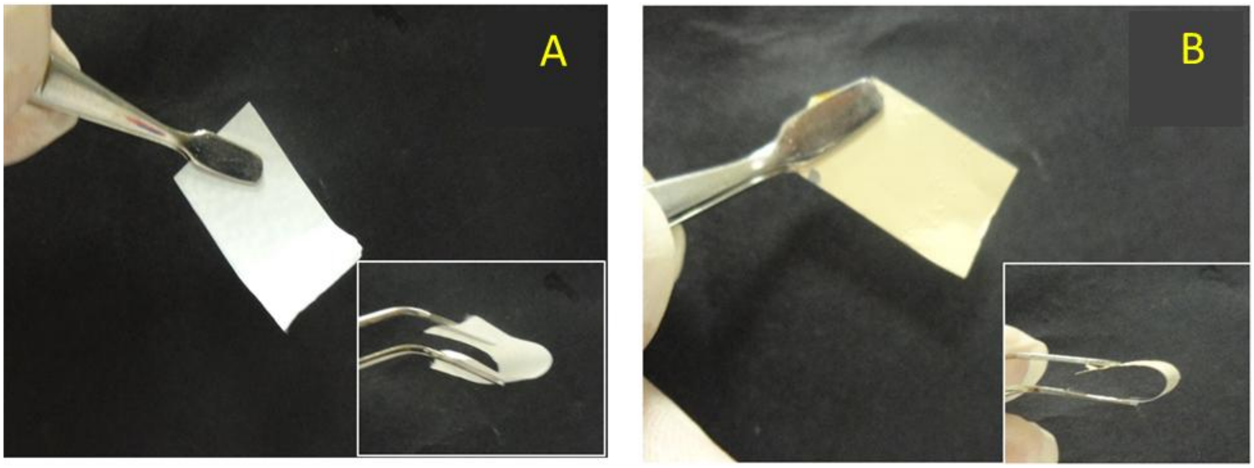

Fig. S4 Representative optical images of (a) as-electrospun PAN nanofibers (NF), (b) TiO_2-PAN NF. Inset show the flexible nature nanofibrous web for the respective samples.

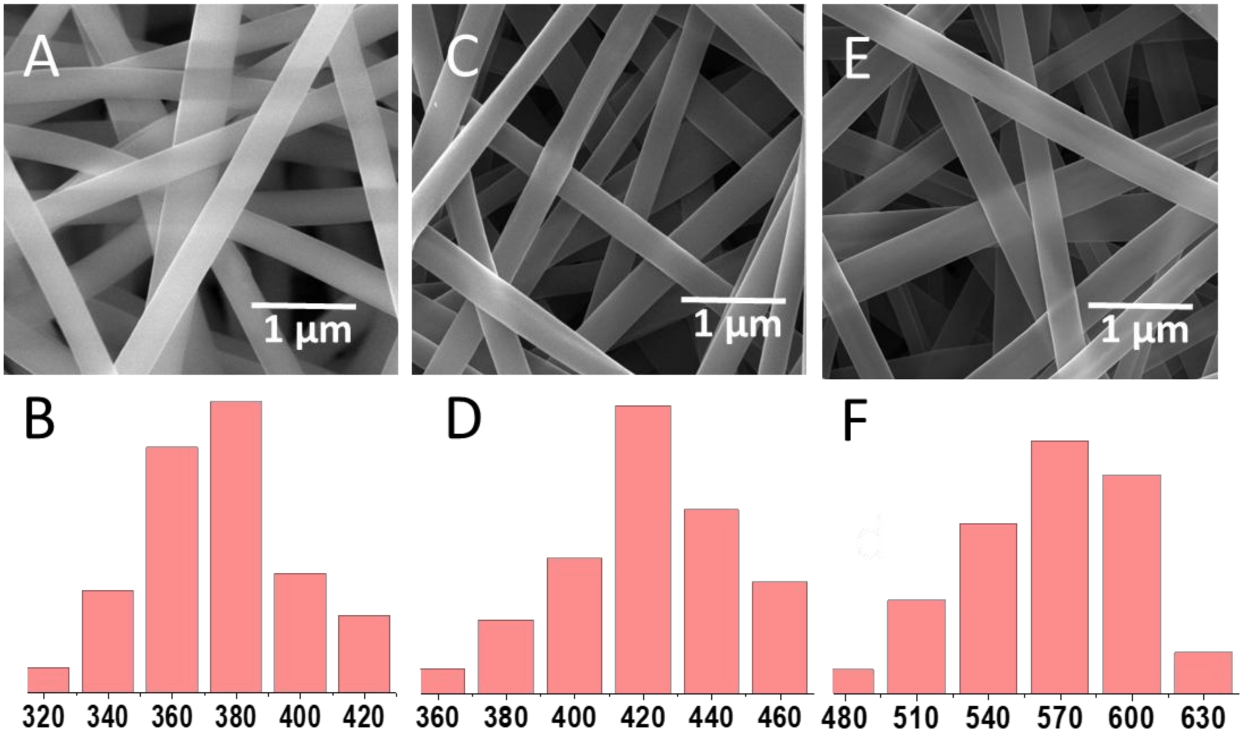

Fig. S5 Representative magnified SEM images and histogram size distribution of (A, B) as-electrospun PAN NF, (C, D) TiO_2-PAN NF and (E, F) Pt-NP/TiO_2-PAN NF.

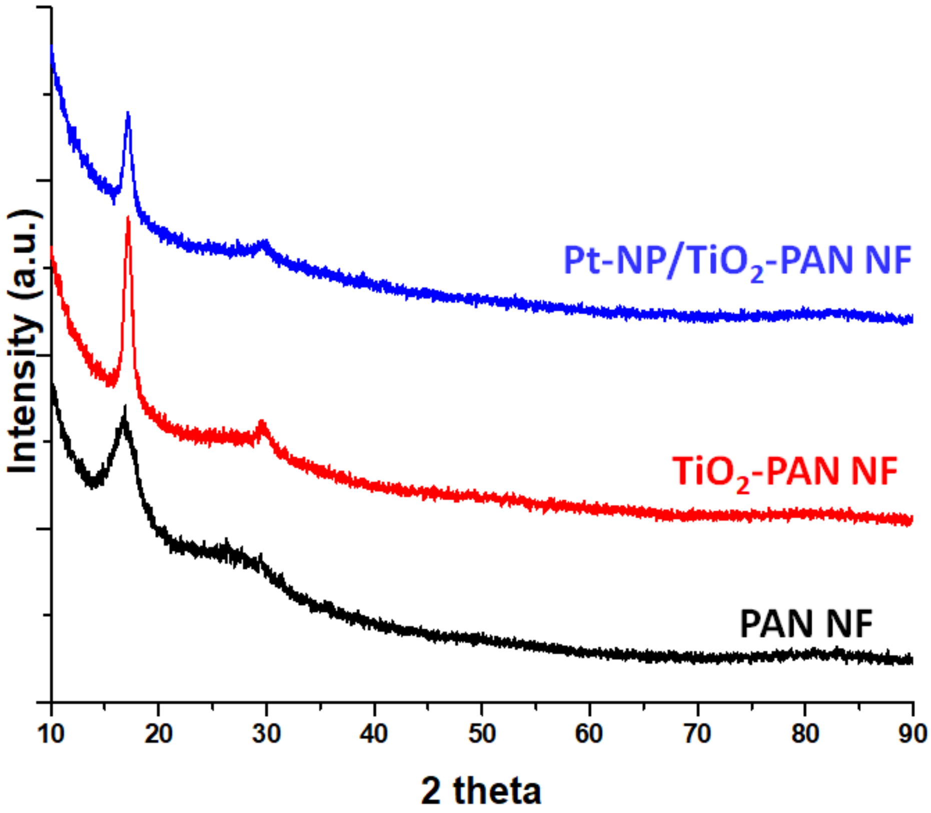

Fig. S6 XRD pattern of the as-electrospun PAN NF, TiO_2-PAN NF and Pt-NP/TiO_2-PAN NF.

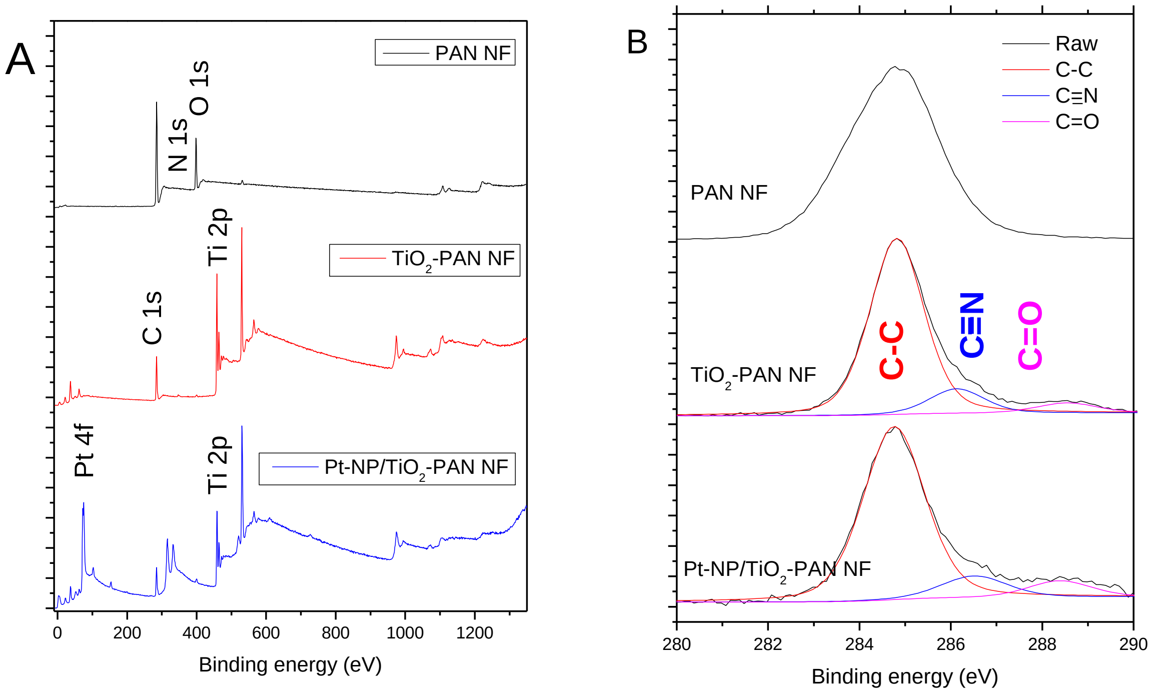

Fig. S7 XPS spectra of as-electrospun PAN NF, TiO_2-PAN NF and Pt-NP/TiO_2-PAN NF: (a) survey; (b) C 1s.

Table S1. XPS survey analysis of TiO_2-PAN NF and Pt-NP/TiO_2-PAN NF.

**Sample**	**Elements**	**Atomic %**
TiO_2-PAN NF	C 1s	37.50
	N 1s	1.49
	O 2s	43.48
	Ti 2p	17.53
	Pt 4f	---
Pt-NP/TiO_2-PAN NF	C 1s	25.70
	N 1s	4.74
	O 2s	49.95
	Ti 2p	11.29
	Pt 4f	8.52

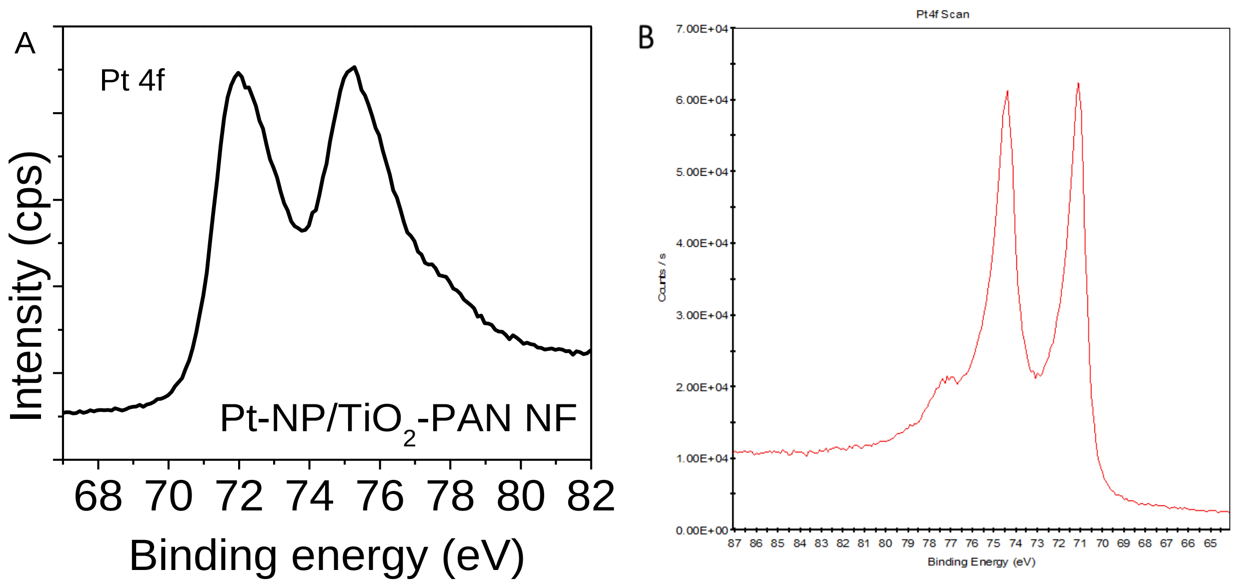

Fig. S8 High resolution Pt 4f XPS spectra of (A) Pt decorated TiO_2-PAN NF and (B) Pt deposited on reference Si substrate under 15 cycles of ALD of Pt.

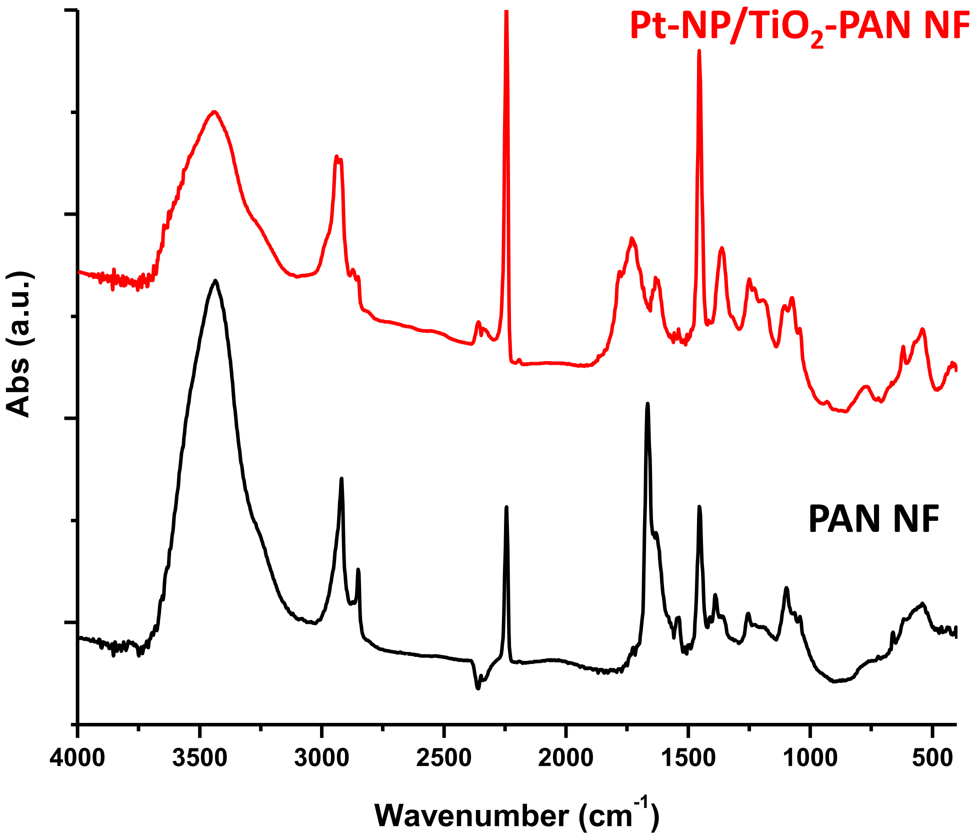

Fig. S9 FTIR spectra of as-electrospun PAN NF and Pt-NP/TiO_2-PAN NF.

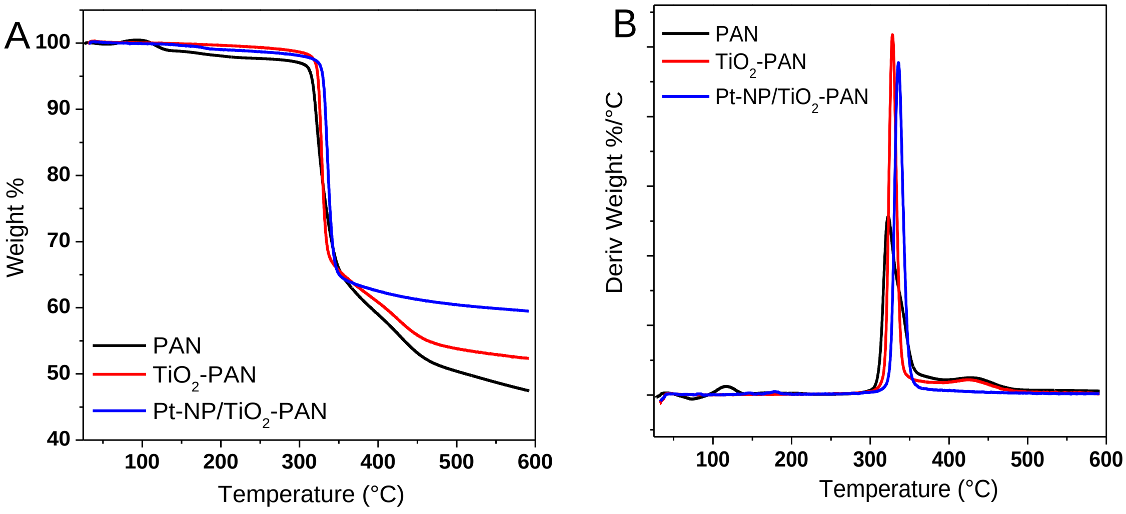

Fig. S10 (A) TGA thermograms and (B) their derivatives of PAN NF, TiO_2-PAN NF and Pt-NP/TiO_2-PAN NF nanofibrous web.

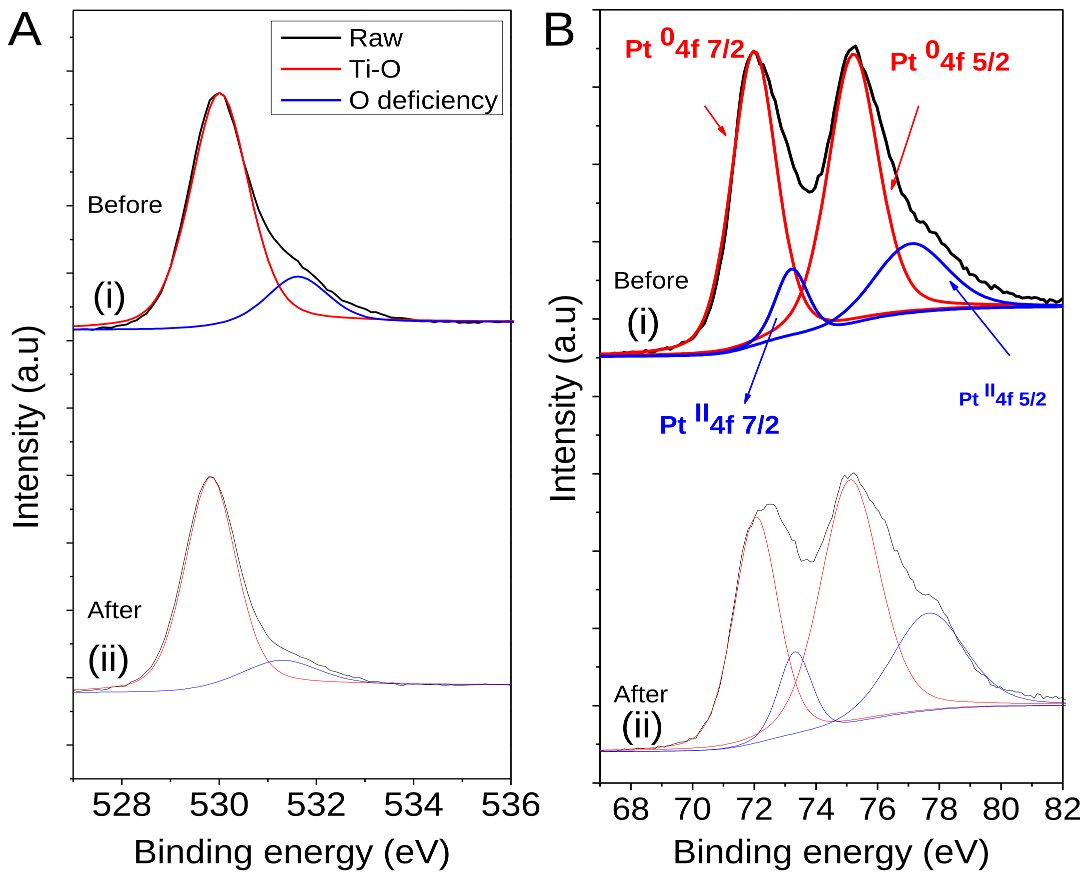

Fig. S11 High resolution of O 2s (A), Pt 4f (B) XPS spectra of Pt-NP/TiO_2-PAN NF nanofibrous web (i) fresh sample (before) (ii) after three consecutive catalytic cycles.
